# Early Postoperative Ondansetron Exposure is Associated with Reduced 90-Day Mortality in Patients Undergoing Cardiac Surgery

**DOI:** 10.3389/fsurg.2022.885137

**Published:** 2022-06-16

**Authors:** Dexin Xiong, Chao Xiong

**Affiliations:** ^1^ Department of Thoracic Surgery, Wuhan Red Cross Hospital, Wuhan, China; ^2^ Department of Anesthesiology, Fuwai Hospital, National Center of Cardiovascular Diseases, Chinese Academy of Medical Sciences, and Peking Union Medical College, Beijing, China

**Keywords:** ondansetron, mortality, acute kidney injury, mimic, cardiac surgery

## Abstract

**Background:**

Ondansetron is a widely used anti-emetic for the prevention and treatment of nausea and vomiting for patients in critical care. Recent retrospective cohort studies suggest the potential beneficial effects of ondansetron in critically ill patients. In this study, we investigate the impact of ondansetron use on patient outcomes after cardiac surgery.

**Material and Methods:**

The MIMIC-III database was used to identify two types of cardiac surgical patients: those who were administered early ondansetron and those who were not given this early medication in the first 48 h in the postoperative period. Multivariable logistic regression was used to investigate the effect of ondansetron exposure on 90-day mortality, acute kidney injury, and malignant ventricular arrhythmias. Sensitivity analyses utilizing the inverse probability of treatment weighting and covariate balancing propensity score models were conducted to test the robustness of our findings.

**Results:**

A total of 12.4% of patients received ondansetron. Ondansetron use was associated with a lower risk of 90-day mortality in the multivariable logistic regression model (OR: 0.31, 95% CI: 0.13 to 0.72; *P* = 0.006) and sensitivity analyses. Additionally, ondansetron exposure was associated with less postoperative acute kidney injury (OR: 0.82, 95%CI: 0.69 to 0.96; *P* = 0.017) but did not increase the risk of postoperative malignant ventricular arrhythmias (OR: 0.38, 95%CI: 0.09 to 1.16; *P* = 0.191).

**Conclusions:**

In a population of cardiac surgical patients, early postoperative use of ondansetron appears to be associated with decreased 90-day mortality and acute kidney injury.

## Introduction

Although the pathophysiology of postoperative nausea and vomiting (PONV) is unclear, the control of PONV through 5-hydroxytryptamine 3 (5-HT3), dopamine, neurokinin 1, and acetylcholine receptor antagonism has become the mainstay of anti-emetic treatment. For cardiac surgical patients, the incidence of PONV is considerable and ranges as high as 43%–69% (1–3). However, PONV prophylaxis is not a widespread routine care in patients undergoing cardiac surgery. Additionally, there is a lack of research on the prophylactic efficacy and safety of anti-emetic drugs in cardiac surgical patients compared with that in other surgical populations.

The 5-HT3 receptor antagonist ondansetron is the most commonly used anti-emetic agent as the “gold standard” in the management of postoperative nausea and vomiting in noncardiac surgical patients (4). In patients undergoing cardiac surgery, ondansetron has also shown prophylactic effect on PONV (1, 3). However, the adverse effect of QT interval prolongation induced by ondansetron administration raises safety concerns about its use in cardiac surgical patients (5–8).

The beneficial effects of 5-HT3 receptor antagonists have also been seen on anti-inflammation (9–12), renal function (13–15), and even mortality reduction (16–20). Recent retrospective studies reported an association between ondansetron use and reduced risk of mortality in critically ill patients both with and without acute kidney injury (AKI) (17–20). Patients undergoing cardiac surgery are also at high risk of developing AKI (21), but research on the mortality benefit of ondansetron use in cardiac surgical patients is lacking.

Therefore, this study aims to determine the association between early postoperative ondansetron use and 90-day mortality in patients undergoing cardiac surgery and whether this association remains in patients with postoperative AKI.

## Materials and Methods

### Setting and Patient Population

This retrospective, observational, single-center cohort study was conducted using data obtained from an open-access database Medical Information Mart for Intensive Care (MIMIC)-III (version 1.4). The MIMIC- III database was approved by The Institutional Review Board (IRB) of the Beth Israel Deaconess Medical Center (2001–P–001699/14). This study was reported following the guidelines of STrengthening the Reporting of OBservational studies in Epidemiology (STROBE).

All adult patients who underwent coronary artery bypass grafting surgery, valve surgery, or both from 2001 to 2012 in the MIMIC-III database were screened. Patients with missing data were excluded from the study. If patients had multiple cardiac surgeries during different hospital stays, only data obtained from these hospital stays for their first cardiac procedure were included.

### Ondansetron Exposure

Postoperative ondansetron use was identified using the inpatient prescription information in the MIMIC-III database. Patients who had ondansetron prescription during the first 48 h after cardiac surgery were considered to be exposed to early postoperative ondansetron. Patients who had ondansetron initiated after 48 h postoperatively but not at the first 48 h after surgery were considered to be not having early postoperative exposure of ondansetron.

### Patient Characteristics

Patients’ baseline and clinical characteristics were collected on the following parameters: age, gender, ethnicity, body mass index (BMI), admission type, surgery type, intraoperative use of cardiopulmonary bypass, comorbidities (congestive heart failure, arrhythmias, pulmonary hypertension, hypertension, peripheral vascular disease, chronic pulmonary disease, diabetes, chronic liver disease, chronic kidney disease, stroke), Sequential Organ Failure Assessment (SOFA) score, and Simplified Acute Physiology Score (SAPS) II. Data in the first 48 h after surgery on the need for renal replacement therapy, use of vasopressors, use of intra-aortic balloon pump (IABP), and prolonged mechanical ventilation defined as ventilation duration longer than 24 h were also collected.

### Study Endpoints

The primary outcome was 90-day mortality defined as death within 90 days after surgery. The secondary outcomes were postoperative AKI and malignant ventricular arrhythmias. Postoperative AKI was defined as AKI within 7 days after surgery and diagnosed by creatinine criteria according to the KDIGO definition. The baseline serum creatinine was defined as the lowest value within 7 days before surgery. If preoperative serum creatinine was not available, the first creatinine measurement during ICU stay was recorded as the baseline value. Postoperative malignant ventricular arrhythmias were defined as the occurrence of ventricular tachycardia, ventricular flutter, or ventricular fibrillation in the postoperative period.

### Statistical Analysis

Continuous variables were checked for normal distribution using the Shapiro–Wilk test. Unpaired Student’s t-test was used for the analysis of parametrically distributed variables. The analysis of nonparametric variables was conducted using the Wilcoxon rank sum test. Continuous variables for individuals with and without ondansetron exposure were presented as mean ± SD or median (interquartile range). Categorical variables for patients treated with and without ondansetron were analyzed using Pearson’s chi-square test or Fisher’s exact test and expressed as number (percentage).

The potential association between early postoperative ondansetron exposure and 90-day mortality was determined by multivariable logistic regression adjusted for covariates including age, gender, ethnicity, BMI, admission type, surgery type, intraoperative use of cardiopulmonary bypass, comorbidities (congestive heart failure, arrhythmias, pulmonary hypertension, hypertension, peripheral vascular disease, chronic pulmonary disease, diabetes, chronic liver disease, chronic kidney disease, and stroke), SOFA score, SAPS II score, postoperative need for renal replacement therapy, use of vasopressors, use of IABP, and prolonged mechanical ventilation. The adjusted odds ratios (ORs) with 95% confidence intervals (CIs) were calculated.

Sensitivity analyses for the primary outcome were conducted to determine the robustness of our findings using the inverse probability of treatment weighting (IPTW) and covariate balancing propensity score (CBPS) models. The IPTW model and the CBPS model were both adjusted by the same covariates included in the logistic model. We also performed a subgroup analysis of the primary outcome in patients having postoperative AKI.

The potential association between early postoperative ondansetron exposure and secondary outcomes including postoperative AKI and postoperative malignant ventricular arrhythmias was determined by multivariable logistic regression. Data were presented as ORs and 95% CIs.

All statistical analyses were performed using R software version 4.1.2, and *P* < 0.05 was considered significant.

## Results

### Patients’ Characteristics

**Figure 1:**
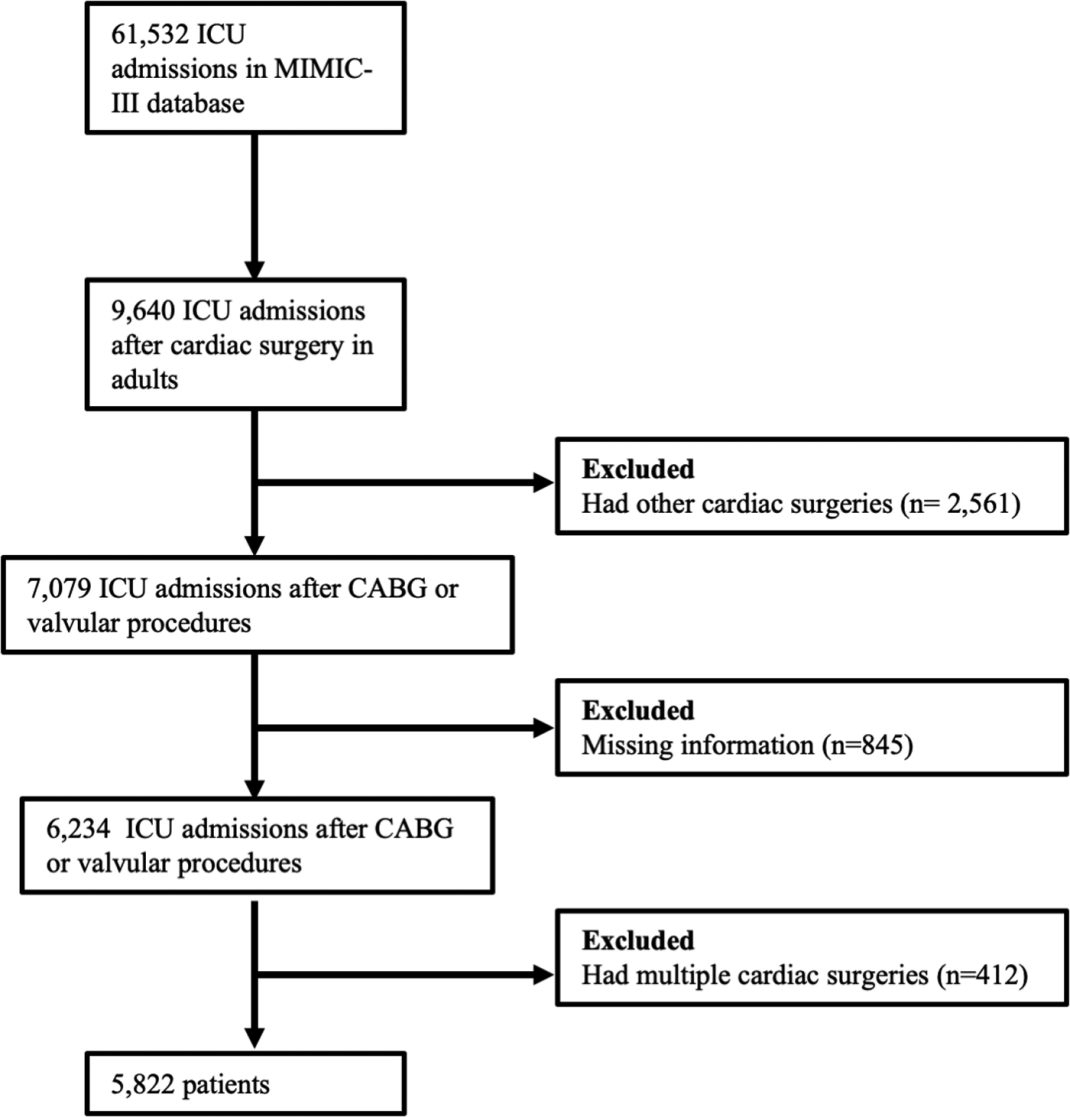
Study cohort inclusion and exclusion flow diagram.

Between 2001 and 2012, the MIMIC-III database reported 9,640 ICU admissions after cardiac surgery for adults. Of these, 5,822 patients who underwent their first CABG and/or valve surgery and without missing data were included in our analysis (Figure 1). Of these, 721(12.4%) had ondansetron exposure in the first 48 h after surgery, and 5,101 (87.6%) had no exposure to ondansetron in this period. Patients’ baseline and clinical characteristics, as well as outcomes categorized by ondansetron exposure, are presented in Table 1. Unadjusted comparisons showed that patients who received early postoperative ondansetron administration were more often male, younger, had a greater body mass index, less non-elective surgery, less congestive heart failure and peripheral vascular disease, lower scores of SOFA and SAPS II, and less intra-aortic balloon pump and prolonged mechanical ventilation compared with those who did not receive early ondansetron.

**Table 1:** Characteristics and outcomes of the study population in the MIMIC–III database according to ondansetron exposure.

	Non-OND group (*n* = 5,101)	OND group (*n* = 721)	*P* value
**Baseline characteristics**			
Age, years	69.9 (59.1–76.4)	66.4 (57.8–74.4)	0.009
Gender			<0.001
male	3,606 (70.7%)	417 (57.8%)	
female	1,495 (29.3%)	304 (42.2%)	
Ethnicity			0.091
White	3,779 (73.9%)	559 (77.5%)	
African American	165 (3.2%)	17 (2.4%)	
Unknown/others	1,166 (22.9%)	145 (20.1%)	
BMI, kg/m^2^	27.9 (24.7–31.7)	27.1 (23.9–31.0)	<0.001
Admission type			<0.001
nonelective	2,710 (53.1%)	291 (40.4%)	
elective	2,391 (46.9%)	430 (59.6%)	
Surgery type			0.014
CABG only	2,820 (55.3%)	374 (51.9%)	
valve only	1,426 (28.0%)	239 (33.2%)	
combined (CABG + valve)	855 (16.8%)	108 (15.0%)	
Intraoperative use of CPB	4,812 (94.3%)	689 (95.6%)	0.177
Congestive heart failure	1,463 (28.7%)	147 (20.4%)	<0.001
Arrhythmias	2,637 (51.7%)	364 (50.5%)	0.543
Pulmonary hypertension	747 (14.6%)	113 (15.7%)	0.466
Hypertension	3,671 (72.0%)	497 (68.9%)	0.091
Peripheral vascular disease	398 (7.8%)	72 (10.0%)	0.044
Chronic pulmonary disease	1,014 (19.9%)	154 (21.6%)	0.270
Diabetes	1,653 (32.4%)	209 (29.0%)	0.065
Chronic liver disease	166 (3.3%)	21 (2.9%)	0.626
Chronic kidney disease	472 (9.3%)	62 (8.6%)	0.569
Stroke	313 (6.1%)	50 (6.9%)	0.406
**Severity of illness**			
SOFA	5 (3–7)	4 (3–6)	<0.001
SAPS II	34 (28–42)	32 (26–40)	0.002
**In the first 2 days after surgery**			
Need of RRT	150 (2.9%)	13 (1.8%)	0.083
Use of vasopressors	4,267 (83.7%)	588 (81.6%)	0.157
Use of IABP	327 (6.4%)	20 (2.8%)	<0.001
Time to extubation >=24 h	589 (11.6%)	19 (2.6%)	<0.001
**Outcomes**			
90-day mortality	186 (3.7%)	6 (0.8%)	<0.001
AKI	2,754 (54.0%)	327 (45.4%)	<0.001
Postoperative malignant	54 (1.1%)	2 (0.3%)	0.044
ventricular arrhythmias			

*Data are presented as number (%) or median (interquartile range). Wilcoxon rank sum and Pearson’s chi-square tests were used to perform univariable analyses for continuous and categorical variables, respectively. BMI*, *body mass index; CABG*, *coronary artery bypass grafting; CPB*, *cardiopulmonary bypass; SOFA*, *Sequential Organ Failure Assessment; SAPS*, *Simplified Acute Physiology Score; IABP*, *intra-aortic balloon pump; AKI*, *acute kidney injury; RRT*, *renal replacement therapy.*

### 90-day Mortality

**Figure 2:**
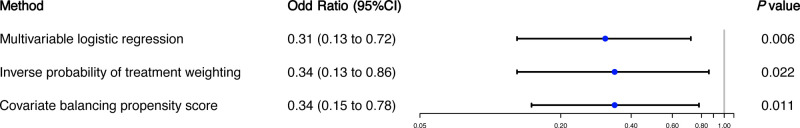
Ondansetron exposure in the first 48 h after cardiac surgery and 90-day mortality. The primary outcome analysis was performing with three different models: (1) multivariable logistic regression; (2) inverse probability of treatment weighting; and (3) covariate balancing propensity score.

The overall 90-day mortality rate was 3.3% (*n* = 192) of 5822 patients in the whole cohort and was significantly lower in patients with ondansetron exposure (6 of 721; 0.8%; *P* < 0.001) than in those without ondansetron exposure (186 of 5101; 3.7%) (Table 1). In multivariable logistic regression analysis, the patients exposed to ondansetron were at a significantly lower risk of 90-day mortality than ondansetron-naïve patients (OR: 0.31, 95% CI: 0.13–0.72; *P* = 0.006) (Figure 2).

In sensitivity analysis using IPTW, there were meaningful differences in the distribution of baseline and clinical characteristics between patients with and without ondansetron exposure. After IPTW, 90-day mortality remained significantly lower in those having early postoperative ondansetron exposure (OR: 0.34, 95%CI: 0.13 to 0.86; *P* = 0.022) (Figure 2). Additionally, we accounted for baseline and clinical characteristics differences using the CBPS model and demonstrated that the result remained significant with an adjusted OR of 0.34 (95%CI: 0.15 to 0.78, *P* = 0.011) for 90-day mortality (Figure 2). In a subgroup analysis of 3,081 patients with postoperative AKI, the association between early ondansetron use and 90-day mortality remained significant in patients with postoperative AKI (OR: 0.34, 95%CI: 0.13–0.87; *P* = 0.025).

### Secondary Outcomes

**Figure 3:**
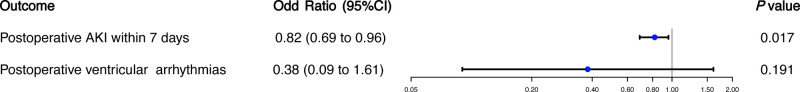
Ondansetron exposure in the first 48 h after cardiac surgery and secondary outcomes. The analyses of secondary outcomes were conducted using multivariable logistic regression analysis.

In the unadjusted comparisons, the incidence of postoperative AKI and sepsis, and malignant ventricular arrhythmias were all significantly lower in patients who had early postoperative ondansetron use than in those not exposed to early ondansetron (Table 1). In multivariable logistic regression analyses, early postoperative ondansetron exposure was associated with a reduced risk of developing postoperative AKI (OR: 0.82, 95%CI: 0.69–0.96; *P* = 0.017), but there was no significant difference in the risk of postoperative and malignant ventricular arrhythmias (OR: 0.38, 95%CI: 0.09–1.16; *P* = 0.191) between the exposed and unexposed patients (Figure 3).

## Discussion

This study showed that early postoperative ondansetron use was associated with a reduced 90-day mortality in patients undergoing cardiac surgery. This association remained consistent in two sensitivity analyses using different methods of covariate adjustment. Furthermore, ondansetron exposure was associated with a significantly reduced risk of 90-day mortality in cardiac surgical patients complicated with postoperative AKI. We further found that ondansetron use was associated with lower risks of both AKI and sepsis in the postoperative period. Although there was concern regarding QT interval prolongation induced by ondansetron, we demonstrated that early ondansetron use after cardiac surgery was not associated with a higher risk of malignant ventricular arrhythmias.

The finding that ondansetron use contributes to reduced mortality aligns with the conclusions of previous studies among critically ill patients. In a retrospective study of a large COVID-19 patient cohort, the use of no less than 8 mg of ondansetron within 48 h of hospital admission was associated with a reduced risk of 30-day mortality for all hospitalized patients (Hazard ratio: 0.55, 95%CI: 0.42–0.70; *P* < 0.001) and those admitted to ICU (Hazard ratio: 0.52, 95%CI: 0.31–0.87; *P* = 0.012) after severe acute respiratory syndrome coronavirus 2 infection (16). In another retrospective cohort study using a large multicenter ICU database, ondansetron received within 24 h after ICU admission was associated with a 5.48% decrease (95%CI: −6.17–−4.79; *P* < 0.001) of 90-day mortality in the regression analysis of the propensity score–matched cohort (17). Additionally, this association was not seen by other anti-emetics including prochlorperazine and metoclopramide, suggesting that the beneficial effect of ondansetron on survival might be related to effects other than anti-emetic (17). Furthermore, our previous study found in the MIMIC-IV database that a low-to-moderate but not a high dose of ondansetron within 48 h after ICU admission was associated with decreased in-hospital mortality in ICU patients (22).

Our study shows that early postoperative ondansetron exposure is associated with better survival at 90 days after surgery in patients with postoperative AKI. This finding is similar to that of three other retrospective cohort studies that investigated critically ills patients with AKI and demonstrated that patients who received ondansetron had significantly lower ICU mortality, in-hospital mortality, and 90-day mortality, respectively (17–19). Additionally, the genes of 5-HT3 receptor were reported to be upregulated in AKI kidneys, and a molecular mechanism study by gene expression signatures demonstrated that the NF-KB and JAK-STAT pathways might be involved in the potential therapeutic effect of ondansetron in critically ill patients complicated by AKI (18). These findings suggest the possibility of the 5-HT3 receptor as a therapeutic target for the treatment of AKI patients.

Additionally, patients exposed to ondansetron had a lower risk of postoperative AKI within 7 days after surgery in our cardiac surgical cohort. But in a similar study, ondansetron exposure within 24 h after ICU admission was not associated with a reduced risk of AKI developed during 25 to 72 h after admission (17). A case-crossover study using a nationwide representative sample of citizens in New Zealand reported even an increased risk of AKI (OR:1.43, 95%CI: 1.25–1.64; *P* < 0.05) for ondansetron users (23). Of note, the renoprotective effect of another 5-HT3 receptor antagonist, tropisetron, has been found in several recent animal studies, probably due to its anti-inflammatory property (13–15). Similarly, ondansetron was reported to exert anti-inflammatory effects in animal models of pancreatitis, colitis, and hepatic injury (10–12). Thus, these findings together suggest that the potential renoprotective effect of ondansetron is worth investigating by further studies.

Prolonged QT interval is common in patients receiving ondansetron, and whether the use of ondansetron increases the risk of fatal ventricular arrhythmias is of great concern (5–8). Our results showed that early ondansetron exposure after cardiac surgery was not associated with an increased risk of malignant ventricular arrhythmias, suggesting the safety of ondansetron use in this population. However, more research needs to be undertaken on the relationship between ondansetron use and fatal arrhythmias.

There were several limitations in our study. First, our study was a retrospective observational investigation. Residual confounding might still exist even if we minimized the effect of confounding using different covariate adjustment methods. Further, we did not calculate the dose of ondansetron because the accurate daily dose of ondansetron administration was not available in the MIMIC-III database. Finally, due to the relative low mortality rate in cardiac surgical patients compared with other patients admitted to ICU, we were unable to perform more subgroup analyses because of the subgroup size.

## Conclusions

Ondansetron was used by 12.4% patients within 48 h after cardiac surgery. The use of ondansetron was associated with a reduced risk of 90-day mortality and AKI within 7 days after surgery. The association between ondansetron use and 90-day mortality remained in patients with postoperative AKI. Additionally, the observed benefit of early ondansetron exposure was without an increased risk of postoperative malignant ventricular arrhythmias. Given the findings of previous studies and our investigation, the use of ondansetron in the early postoperative period after cardiac surgery should be considered.

## Data Availability

The raw data supporting the conclusions of this article will be made available by the authors, without undue reservation.
